# Signature-Tagged Mutagenesis in a Chicken Infection Model Leads to the Identification of a Novel Avian Pathogenic *Escherichia coli* Fimbrial Adhesin

**DOI:** 10.1371/journal.pone.0007796

**Published:** 2009-11-12

**Authors:** Esther-Maria Antão, Christa Ewers, Doreen Gürlebeck, Rudolf Preisinger, Timo Homeier, Ganwu Li, Lothar H. Wieler

**Affiliations:** 1 Institut für Mikrobiologie und Tierseuchen, Freie Universität Berlin, Berlin, Germany; 2 Lohmann Tierzucht GmbH, Cuxhaven, Germany; 3 Department of Veterinary Microbiology and Preventive Medicine, College of Veterinary Medicine, Iowa State University, Ames, Iowa, United States of America; 4 Institute of Animal Hygiene and Veterinary Public Health, Veterinary Faculty, Universität Leipzig, Leipzig, Germany; Universita di Sassari, Italy

## Abstract

The extraintestinal pathogen, avian pathogenic *E. coli* (APEC), known to cause systemic infections in chickens, is responsible for large economic losses in the poultry industry worldwide. In order to identify genes involved in the early essential stages of pathogenesis, namely adhesion and colonization, Signature-tagged mutagenesis (STM) was applied to a previously established lung colonization model of infection by generating and screening a total of 1,800 mutants of an APEC strain IMT5155 (O2:K1:H5; Sequence type complex 95). The study led to the identification of new genes of interest, including two adhesins, one of which coded for a novel APEC fimbrial adhesin (Yqi) not described for its role in APEC pathogenesis to date. Its gene product has been temporarily designated ExPEC Adhesin I (EA/I) until the adhesin-specific receptor is identified. Deletion of the ExPEC adhesin I gene resulted in reduced colonization ability by APEC strain IMT5155 both *in vitro* and *in vivo*. Furthermore, complementation of the adhesin gene restored its ability to colonize epithelial cells *in vitro*. The ExPEC adhesin I protein was successfully expressed *in vitro*. Electron microscopy of an afimbriate strain *E. coli* AAEC189 over-expressed with the putative EA/I gene cluster revealed short fimbrial-like appendages protruding out of the bacterial outer membrane. We observed that this adhesin coding gene *yqi* is prevalent among extraintestinal pathogenic *E. coli* (ExPEC) isolates, including APEC (54.4%), uropathogenic *E. coli* (UPEC) (65.9%) and newborn meningitic *E. coli* (NMEC) (60.0%), and absent in all of the 153 intestinal pathogenic *E. coli* strains tested, thereby validating the designation of the adhesin as ExPEC Adhesin I. In addition, prevalence of EA/I was most frequently associated with the B2 group of the EcoR classification and ST95 complex of the multi locus sequence typing (MLST) scheme, with evidence of a positive selection within this highly pathogenic complex. This is the first report of the newly identified and functionally characterized ExPEC adhesin I and its significant role during APEC infection in chickens.

## Introduction

Research on avian pathogenic *E. coli* (APEC) has increased greatly over the years where the pathogen has been known to cause disease among chickens and other fowls which usually results in large economic losses for the poultry industry [1]. APEC are mostly associated with infection of extraintestinal tissues in chickens, turkeys, ducks and other avian species. The most important disease syndrome associated with APEC begins as a respiratory tract infection and may be referred to as aerosacculitis or the air sac disease [2]. This inevitably results in severe systemic infection leading to death of the animal infected. These pathogens have recently gained even more importance, now being classified under the category of extraintestinal pathogenic *E. coli* (ExPEC), a group which includes both human pathogens like the uropathogenic *E. coli* (UPEC) and newborn meningitic *E. coli* (NMEC) and animal pathogens, which in turn suggest the possibility of APEC having zoonotic potential [3]–[5].

Microbial pathogenicity is a complex phenomenon encompassing diverse mechanisms. There are, however, several common strategies that pathogenic organisms use to sustain themselves and overcome host barriers, one of them being the firm adhesion of the micro-organism to host cells [6]. Colonization is crucial to pathogenesis of bacteria, being the earliest stage during onset of the disease and the ability to adhere to host surfaces is by far the most vital step in the successful colonization by microbial pathogens [7], [8]. The presence of adhesins are said to be essential to the first steps of bacterial pathogenicity [9]. A distinct family of adhesins called the adhesive pili or fimbriae, encoded by adhesin-gene clusters and assembled by the chaperone-usher pathway are said to be ubiquitous in Gram-negative organisms [10]. Some of the well known fimbriae present among ExPEC strains are Type I fimbriae (*fim*), P fimbriae or the pilus associated with pyelonephritis (*pap*), curli fibre (*csg*), S fimbriae or the sialic acid-specific fimbriae (*sfa*), F1C fimbriae (*foc*), afimbrial adhesin/Dr antigen-specific adhesin (*afa*/*dra*), heat-resistant agglutinin (*hra*) and others [4]. Each of these adhesins recognizes a specific receptor although as a group they share common genomic organization, assembly and even quaternary structural traits [10].

The role of some of these adhesins during APEC infection is well recognized. It has been reported that bacterial colonization of the respiratory tract is mediated by fimbrial adhesins, and studies have shown that type 1 and P fimbriae are expressed *in vivo* by bacteria colonizing the lung, air sacs and internal organs of chickens during infection [11]. Bacterial adhesion to pharyngeal epithelial cells of chickens was also found to be inhibited by the presence of D-mannose, demonstrating the role of type 1 fimbriae during the early stages of the disease [12]. No expression of P fimbriae was seen in the chicken trachea, suggesting that they may be important only in later stages of infection [11]. In another study, it was seen that 99% of *E. coli* isolated from diseased birds possessed the *csgA* gene responsible for curli biosynthesis [2]. Furthermore curli fibres were found to be essential for the internalization of bacteria causing avian septicaemia as seen *in vitro* [13].

Although there is increased knowledge about fimbrial adhesins and other colonization factors including the role they might play during infection, further investigation is still required to completely elucidate the function of each adhesin and its specific role in pathogenesis. In addition, the availability of complete genome sequences for some ExPEC pathogens like UPEC strain UTI89 (O18:K1:H7) and APEC strain APEC_O1 (O1:K1:H7) has drawn our attention to the large increase in the number of genes with unknown function, possibly involved in adhesion and colonization, and whose contribution to virulence on the whole is presently merely hypothetical [14]–[16]. Determination of the function of these novel genes will lead to a deeper understanding of host-pathogen interactions during the initial stages of APEC infection.

Previously our laboratory applied Signature-tagged mutagenesis (STM) to APEC in a chicken systemic infection model which led to the successful identification of genes crucial to systemic infection in chickens, however no adhesins were identified [17]–[20].

In the present study, we applied STM to APEC in a modified lung infection model [21], in order to identify novel genes functionally involved in the adhesion and colonization of the chicken lung during the infection process. The STM screen led, among others, to the identification of a mutant EA7F9 with a transposon insertion in a putative adhesin gene (*yqi*). In addition, a mutant with an insertion in a gene encoding the type 1 fimbriae regulatory protein was also identified. These two genes were the most obvious targets of the STM screen being adhesins, the primary structures in direct contact with host tissue during infection. Type 1 fimbriae are already well characterized for their role in ExPEC pathogenesis; however, *yqi* has until now never been described for its potential role during infection. Therefore, the new adhesin encoded by the gene *yqi* became the target of this study, and was further characterized for its role in the initiation of APEC infection. We describe for the first time, the identification and characterization of this novel adhesin in APEC, temporarily renamed ExPEC adhesin I (EA/I), and the essential role it plays in the initial colonization of the chicken lung during infection.

## Results

### Attenuation of IMT5155 with Mutation in ExPEC Adhesin I (EA/I) Gene *yqi*

**Figure 1:**
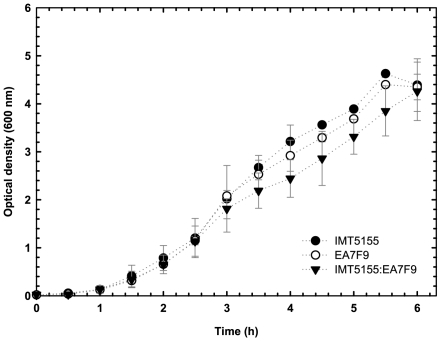
Graphic illustrating growth of transposon mutant EA7F9 in competition with IMT5155 and separately in Luria Bertani (LB) growth medium. A competition index (CI) *in vitro* was calculated at a time point of 4 h.

Among the many genes identified during the STM screen, was the ExPEC adhesin I (EA/I) gene, also annotated as *yqi* [15]. *In vitro* competition assays of transposon-mutant EA7F9, with a mutation in gene *yqi*, versus wild type strain IMT5155 showed that there was no significant growth defect *in vitro* as determined by growth curves (Figure 1). An *in vitro* competition index of 1.2 was observed for EA7F9. On the other hand, the mutant was found to be attenuated in the chicken with an *in vivo* competition index of 0.6. This result confirmed the attenuation of mutant EA7F9 during STM screening.

### Sequencing of the EA/I Gene Cluster Region in IMT5155

**Figure 2:**
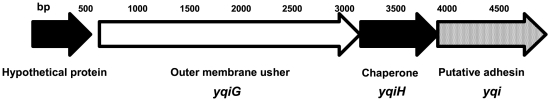
Physical map showing genomic organization of the 4,975 bp *yqi* adhesin gene cluster in APEC strain IMT5155. A hypothetical protein preceeds the putative outer membrane usher protein, followed by the putative chaperone and finally the putative adhesin.

The putative *yqi* adhesin gene cluster was completely sequenced in APEC strain IMT5155. Genomic organization of the IMT5155 *yqi* adhesin gene cluster was as follows: the putative outer membrane usher preceded the periplasmic chaperone, followed by the adhesin gene. A conserved hypothetical protein gene precedes the usher gene which may code for the adhesin subunit protein although this has not yet been confirmed (Figure 2). This 4,975 bp region showed a hundred percent sequence identity with the UPEC and APEC *yqi* adhesin gene cluster in sequenced strains UTI89 (Acc. No: CP000243) and APEC_O1 (Acc. No: CP000468) [15]. Comparing the sequence with 1096 bacterial genomes currently available in the public database, out of which 28 were *Escherichia coli* genomes, this adhesin gene cluster is only found among ExPEC strains including APEC, UPEC and NMEC and is not harboured by intestinal pathogenic *E. coli* like enteropathogenic *E. coli* (EPEC) or enterohaemorrhagic *E. coli* (EHEC) such as *E. coli* O157:H7 strains EDL933 and Sakai, or non pathogenic *E. coli* K-12 strains MG1655 and W3110.

### EA/I Plays a Role in the Adhesion of APEC to Chicken Fibroblasts and Kidney Epithelial Cells *In Vitro*

**Figure 3:**
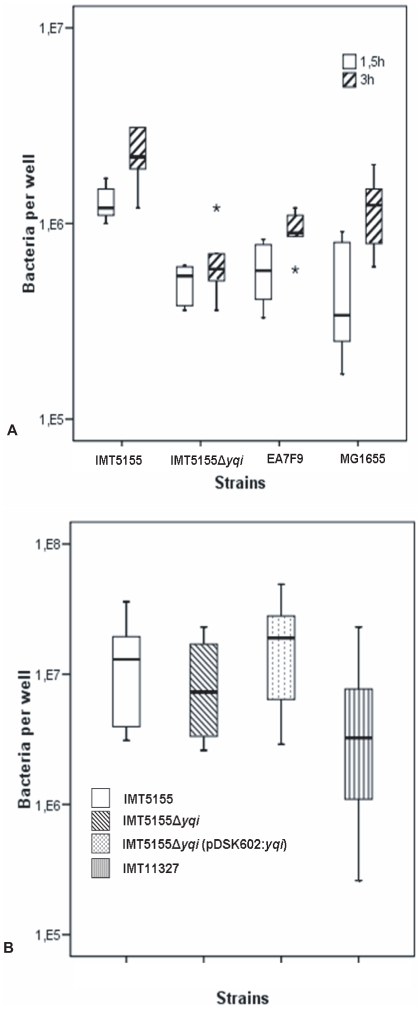
Bacterial adhesion to chicken fibroblast cells 1.5 h and 3 h after infection with an MOI = 100. Differences between IMT5155 and IMT5155Δ*yqi* were statistically significant with a *p*<0.005 at 1.5 h and *p*<0.05 at 3 h (A). Bacterial adhesion to polarized Madin Darby canine kidney (MDCK-1) cells 3 h after infection with an MOI = 100. The difference between IMT5155Δ*yqi* and IMT5155Δ*yqi* (pDSK602:*yqi*) was significant with a *p*<0.04 (B).

In order to determine the effect of EA/I in APEC, adhesion assays were initially performed *in vitro* using chicken fibroblast cells. Strain EA7F9 with a disruption in the *yqi* gene via a transposon (STM generated mutant), and strain IMT5155Δ*yqi* devoid of the ExPEC adhesin I gene were tested against the wild type pathogen IMT5155 and a negative control strain MG1655. Bacterial determination at two different time points revealed a reduction in the ability of both EA7F9 and IMT5155Δ*yqi* to adhere to fibroblast cells up to about forty percent of the total adhesion by IMT5155 (Figure 3A). MG1655 as expected also showed decreased adhesion ability when compared with IMT5155. An average CFU was calculated from three independent wells, and results were reproducible between adhesion experiments. Statistical analysis of the CFU values showed a significant difference between wild type IMT5155 and mutants tested, with a *p*<0.005 and *p*<0.05 at 1.5 h and 3 h respectively (Figure 3A).

To further confirm the role of *yqi* in APEC *in vitro*, adhesion assays were carried out using polarized Madin-Darby Canine-Kidney (MDCK-1) epithelial cells. Strains IMT5155Δ*yqi* and IMT5155Δ*yqi* (pDSK602:*yqi*), a strain carrying a modified expression plasmid with the *yqi* gene, that is, the complemented mutant, were tested against IMT5155, the positive control, and IMT11327, a negative control strain of avian origin lacking the gene *yqi*. A reduction in colonization by IMT5155Δ*yqi* was seen, up to twenty percent of the total adhesion by IMT5155, 3 h after infection (Figure 3B). The complemented strain IMT5155Δ*yqi* (pDSK602:*yqi*) regained its ability to adhere to MDCK-1 cells by introduction of the *yqi* gene when compared with its deletion mutant IMT5155Δ*yqi* with a significance of *p*<0.04. All results were reproducible in consecutive adhesion experiments.

### Effect of EA/I in Colonization of the Chicken Lung *In Vivo*

**Figure 4:**
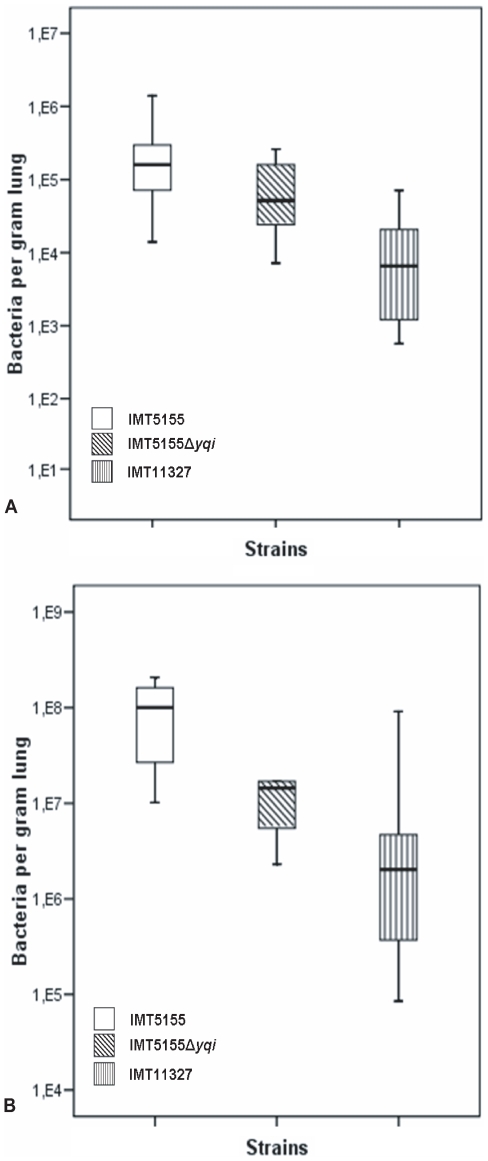
Bacterial colonization of the chicken lungs 24 h after intra-tracheal infection with 10^6^ CFU of bacteria. Differences between IMT5155 and IMT5155Δ*yqi* were statistically significant with a *p*<0.05 (n = 6). Strain IMT11327 is the negative control (A). Bacterial colonization of the chicken lungs 24 h after intra-tracheal infection with 10^9^ CFU of bacteria. Differences between IMT5155 and IMT5155Δ*yqi* were statistically significant with a *p*<0.02 (n = 6). Strain IMT11327 is the negative control (B).

Colonization of the chicken lung *in vivo* was studied by infecting 5-week old chickens intra-tracheally, and isolating bacteria from the lungs 24 h after infection. For this purpose, two different infection set ups were made use of, including the lung colonization model of infection and the systemic infection model. IMT5155 and IMT11327 served as positive and negative controls respectively. The results of the *in vivo* experiments confirmed the observations made *in vitro* in cell culture models described above. When chickens were infected with a dose of 10^6^ CFU of IMT5155Δ*yqi* in a model used to study colonization abilities of the chicken lung by various strains, a distinct reduction in re-isolated bacterial numbers was observed as compared to IMT5155 as depicted in figure 4A. Differences between strains were statistically significant with a *p*<0.05. The average lung score in chickens infected with IMT5155Δ*yqi* was 1.63 in comparison to 1.71 and 1.21 in chickens infected with IMT5155 and IMT11327 respectively. All chickens infected did not exhibit any clinical symptoms.

Moreover, when chickens were infected with a higher dose of bacteria, that is, 10^9^ CFU, in a model designed to induce systemic infection, there still was a significant difference in the colonization of the lung by strains IMT5155 and IMT5155Δ*yqi* as seen in figure 4B with a *p*<0.02 [20]. The average lung score in chickens infected with IMT5155Δ*yqi* was found to be 1.52 in comparison to 2.4 and 0.6 in chickens infected with IMT5155 and IMT11327 respectively. In both infection models, the ability of IMT11327 to colonize the chicken lung was much less than both IMT5155 and IMT5155Δ*yqi* as seen in figure 4.

### Effect of EA/I during Systemic Infection in Chickens

**Figure 5:**
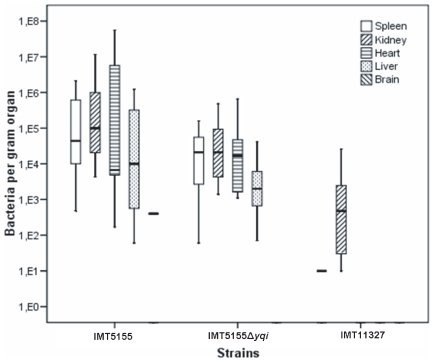
Bacterial re-isolation of IMT5155, IMT5155Δ*yqi* and IMT11327 from the lungs, spleen, liver, heart, kidneys and brain 24 h after intra-tracheal infection with 10^9^ CFU of bacteria (n = 6). Absence of columns indicates that no bacteria were isolated from the organ.

Systemic infection can be induced in chickens by infecting 5-week old birds intra-tracheally with a pathogenic strain with an infection dose of 10^9^ CFU. As mentioned before, infection of chickens with this infection dose resulted in significant reduction in bacterial colonization of the chicken lung. When bacteria were re-isolated from chicken internal organs including spleen, kidneys, heart, liver and brain, there was a reduction in bacterial numbers when infected with IMT5155Δ*yqi* as compared to IMT5155 as seen in figure 5, however only marginal. The only exception was the brain, where almost no bacteria were isolated when infected with IMT5155Δ*yqi* as compared to IMT5155. All organ scores were considerably reduced in chickens infected with IMT5155Δ*yqi* as compared to chickens infected with IMT5155 as seen in table 1.

**Table 1:** Score values for severity of organ lesions ± standard deviation in respiratory and other organs.

Strain	Air sacs	Lung	Liver	Heart	Spleen
**IMT5155**	2.0±1.0	2.4±0.9	0.8±0.8	1.8±1.3	1.0±0.0
**IMT5155Δ** ***yqi***	1.1±0.7^*^	1.5±0.6^*^	0.1±0.3^*^	0.7±0.8	0.9±0.3
**IMT11327**	1.6±0.5^*^	2.0±0.5^*^	0.0±0.0^*^	0.0±0.0	0.9±0.5

Infection with IMT5155, IMT5155Δ*yqi* and IMT11327 at infection dose 10^9^ CFU. Differences in organ scores between IMT5155 and IMT5155Δ*yqi* and between IMT5155 and negative control IMT11327 were statistically significant for air sacs, lungs and liver with a *p*<0.05^*^.

### Electron Microscopy Reveals Fimbrial-Like Appendages Associated with ExPEC Adhesin I (*yqi*) Gene Cluster

**Figure 6:**
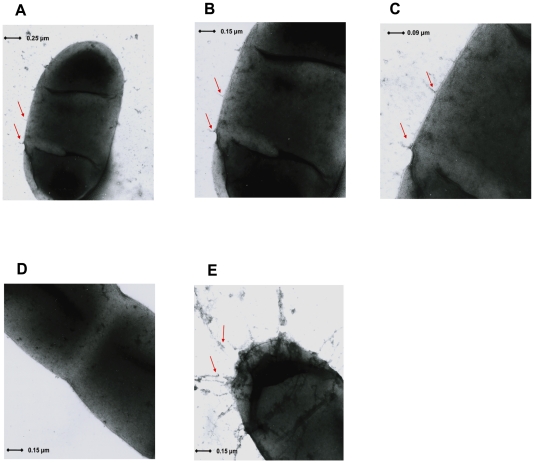
Expression of the ExPEC adhesin I (*yqi*) gene cluster *in vitro*. Electron micrographs show negatively stained afimbriate strain *E. coli* AAEC189 (pKESK:*yqi*_4975_XB) over-expressed with the *yqi* adhesin gene cluster at a magnification of 45,000x, 65,000x and 100,000x (A–C), negative control afimbriate strain *E. coli* AAEC189 (D) and wild type fimbriated *E. coli* strain IMT5155 (E). The arrows indicate the location of the fimbriae.

The ExPEC adhesin I (*yqi*) 4,975 bp gene cluster coding for the putative subunit, chaperone, usher and adhesin was successfully cloned and over-expressed in an afimbriate strain *E. coli* AAEC189. Negative staining of strain AAEC189 (pKESK:*yqi*_4975_XB) revealed the expression of short fimbrial like appendages forming on the outer membrane of the bacterial cell (Figure 6A–C). These appendages were about 0.04 µm long and 0.005 µm thick and were not detected in the afimbriate strain AAEC189, that is, the negative control (Figure 6D). The wild type strain IMT5155, harbouring other adhesins like Type 1 fimbriae and curli, in addition to ExPEC adhesin I, was used as a positive control for the staining method, and long fimbriae with a length of about 0.5 µm (Figure 6E) were observed which were morphologically different from the fimbrial structures observed in strain AAEC189 (pKESK:*yqi*_4975_XB).

### Prevalence of EA/I among ExPEC Reveals Its Potential as a Factor of Virulence

A collection of ExPEC strains, intestinal pathogenic *E. coli*, and avian and human non pathogenic strains available at the Institute of Microbiology and Epizootics, Freie Universität Berlin, was screened to determine the presence of the *yqi* gene among these strains. Out of a total of 588 ExPEC isolates tested for the presence of *yqi*, including 406 APEC, 138 UPEC, 25 NMEC and 19 Septicaemia associated *E. coli* (SePEC), 368 isolates were found to be positive for *yqi*, which amounts to a total of 62.5% of pathogenic isolates harbouring the *yqi* gene (Table 2). Among APEC isolates alone, 221 isolates were found to be positive making it a total of 54.4% positive for *yqi* in this group. Among UPEC isolates 91 isolates were found to be positive in a total percentage of 65.9% positive for *yqi* and among NMEC isolates 15 were found to be positive amounting to 60.0% positive for *yqi*. Finally, among SePEC isolates, 10 were positive in a percentage of 52.6%. From a total of 159 non pathogenic isolates tested, including those from avian and human origin, 31 were found to be positive for *yqi*, which accounts for only 19.4% of the non pathogenic strains tested.

**Table 2:** Prevalence of ExPEC adhesin I coding gene *yqi* among pathogenic *E. coli* strains and non-pathogenic strains of human and avian origin.

E. coli Group	Isolates tested	Prevalence of yqi (%)
APEC	406	54.4
UPEC	138	65.9
NMEC	25	60.0
SePEC	19	52.6
STEC	49	0.0
EHEC	46	0.0
EPEC	28	0.0
aEPEC	12	0.0
ETEC	8	0.0
EIEC	6	0.0
EAEC	4	0.0
Commensals of human and avian origin	159	19.4

APEC = Avian pathogenic *E. coli*, UPEC = Uropathogenic *E. coli*, NMEC = Newborn meningitic *E. coli*, SePEC = Septicaemia associated *E. coli*, STEC = Shiga Toxin-producing *E. coli*, EHEC = Enterohaemorrhagic *E. coli*, EPEC = Enteropathogenic *E. coli*, aEPEC = Atypical Enteropathogenic *E. coli*, ETEC = Enterotoxigenic *E. coli*, EIEC = Enteroinvasive *E. coli*, EAEC = Enteroggregative *E. coli*.

Results were compared with MLST/EcoR data deposited in the publicly available database (www.mlst.net), of which 292 of the pathogenic isolates tested were known for their sequence type (ST) and 607 were known for their EcoR groups according to Herzer et al [22], which was carried out as part of a separate study at the Institute of Microbiology and Epizootics. Of the isolates positive for *yqi*, 68 isolates belonged to the EcoR group A (18.9%), 4 to B1 (1.1%), 255 to B2 (70.8%) and 33 to D (9.1%) making B2 the predominant group for isolates harbouring *yqi*. Among isolates negative for *yqi*, 122 isolates belonged to EcoR group A (49.3%), 29 to B1 (11.7%), 42 to B2 (17.0%) and 54 to D (21.8%) where A is the predominant group for isolates lacking *yqi*.

Out of the 31 non pathogenic isolates found to be positive for *yqi*, 24 (77.4%) were found to belong to EcoR group B2. Additionally 17 of the non pathogenic strains positive for *yqi* were isolated from the intestinal tract of healthy humans. These strains were positive for K1 capsule antigen and also belonged to EcoR group B2.

When comparing the distribution of the *yqi* gene among ExPEC strains with available MLST data, it was observed that strains positive for *yqi* are mostly allotted to sequence types 12, 68, 73, 95, 104, 140, 141, 349, 355, 358, 372, 390, 420, 913. All strains belonging to sequence types 141, 372 and sequence type complex 95 were positive for *yqi*.

Among 153 intestinal pathogenic *E. coli* tested in this study, none of the isolates were found to be positive for *yqi* as seen in table 2.

Due to the exclusive association of the putative fimbrial adhesin gene *yqi* with extraintestinal pathogenic *E. coli* (ExPEC), that is, its high prevalence among ExPEC isolates compared with non pathogenic *E. coli*, and complete absence in intestinal pathogenic *E. coli*, the adhesin was temporarily designated ExPEC adhesin I (EA/I).

### EA/I Is Evolving under Positive Selection

The ExPEC adhesin I gene (*yqi*) was sequenced in many strains and compared with existing multilocus sequence typing (MLST) data. MLST is a nucleotide sequence based approach for the unambiguous characterization of isolates of bacteria using sequences of internal fragments of seven house-keeping genes [23]. The population structure of microbial species with intermediate levels of recombination can thus be revealed by allele-based analyses [23]. Wirth et al. described that sequence polymorphisms could define unique sequences for each of the seven house-keeping gene loci, which are referred to as alleles and each unique combination of alleles is assigned a sequence type (ST) number [24]. Related STs are assigned to so-called ST complexes, using the principles of the eBurst algorithm [25]: each ST complex includes at least three STs that differ from their nearest neighbour by no more than two of the seven loci while ST complexes differ from each other by three or more loci, and STs that did not match the criteria for inclusion within an ST complex are simply referred to by their ST designation [24].

**Figure 7:**
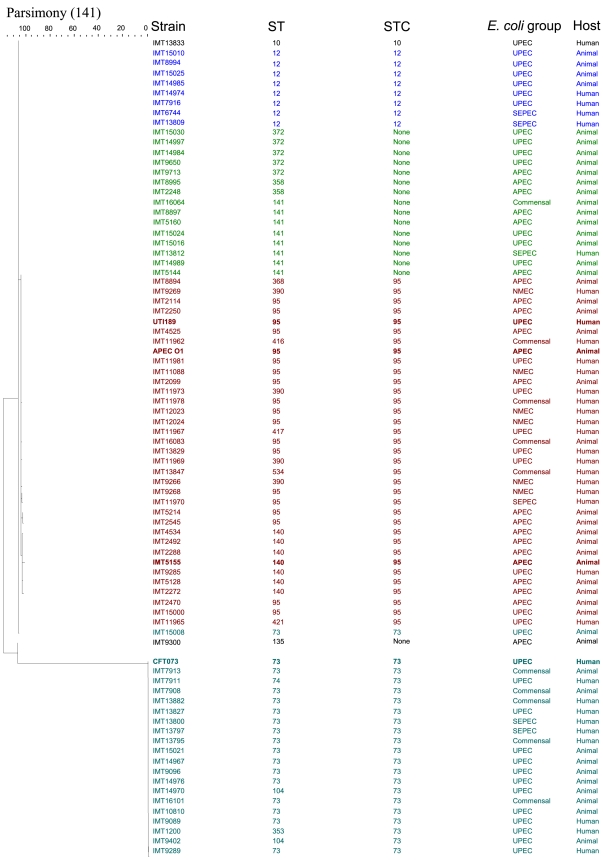
Phylogenetic analysis of ExPEC adhesin I (*yqi*). Maximum Parsimony tree shows distances between *yqi* gene sequences among strains belonging to different sequence types (ST: Sequence type; STC: Sequence type complex).

The evolutionary history of the *yqi* gene was inferred using the Maximum Parsimony method. Sequencing of the ExPEC adhesin I (*yqi*) gene in strains belonging to the various sequence types (ST) and sequence type complexes (STC) including STC10, STC12, STC73, STC95, ST141 and ST372, and calculation of the distances of the adhesin gene locus between these strains revealed distribution of the strains tested into two groups (Figure 7). The value of replicate trees in which the associated taxa clustered together in the bootstrap test was 99% for the two groups identified. One group included strains belonging to the ST73 complex, while all other strains were assigned to a second group. Sequence homology was observed among strains belonging to a particular sequence type or sequence type complex as is the case with ST141, ST372, STC12 and STC73 within the sequence type or sequence type complex (Figure 7). One exception to the rule was strain IMT15008, ST73, STC73 which showed variations in its *yqi* gene sequence compared to other strains in the ST73 complex, and could be better assigned to the group harbouring other strains tested. Interestingly, only strains belonging to sequence type complex 95 showed mutations in the *yqi* gene sequence. Therefore, we determined the ratio of the non-synonymous mutation rate (Dn) to the synonymous mutation rate (Ds) within this complex using DnaSP 4.50.3 software. Dn/Ds < or  = 1 indicates purifying or neutral selection, favouring amino acid substitutions [26]. Our data show a non-synonymous mutation rate of 0.00095496 and a synonymous mutation rate of 0.00024 resulting in a Dn/Ds ratio of 3.979, indicating strong positive selection for structural evolution of the adhesin gene *yqi* within the sequence type 95 complex.

## Discussion

A necessary step in the successful colonization and progression of disease by microbial pathogens is the ability to adhere to host surfaces [8]. The present study made use of a lung colonization chicken infection model in order to identify APEC genes specific to early stages of infection including adhesion to and colonization of the host by its pathogen via signature-tagged mutagenesis. As anticipated, we identified fimbrial proteins including the type 1 fimbrial regulatory protein, and other novel genes explicitly involved in the early stages of APEC pathogenesis as determined by competition assays with mutants. Our most interesting finding was a novel *yqi* adhesin gene which was seen to play a very significant role in the colonization of the avian lung by APEC. The newly identified putative fimbrial adhesin encoded by gene *yqi*, will be temporarily called ExPEC adhesin I (EA/I) until the specific host receptor for this adhesin has been identified in order to allow better classification and nomenclature of the novel *E. coli* fimbrial adhesin.

Adhesins are known to facilitate host colonization by mediating the first and crucial interaction with host tissue [10]. In certain cases pathogens have been reported to have a substantial amount of different adhesins expressed at one time or another [8]. In *E. coli*, two of the best characterized adhesins are the type 1 fimbriae and the P fimbriae which are regulated by the *fim* and *pap* operons [27]. Genome sequencing of prototypic cystitis UPEC strain UTI89 has now revealed ten different chaperone-usher adhesin systems, among which the *auf*, *yad*, *yfc*, *yqi*, *yeh* and *fml* operons still remain to be characterized [14].

Interestingly, we identified the UPEC *yqi* adhesin gene in an STM screen specially designed to detect factors that would play a crucial role in the initiation of APEC infection. As stated before, the APEC *yqi* gene showed complete sequence identity with the UPEC *yqi* and is part of a 4,975 bp adhesin gene cluster yet uncharacterized. When comparing this adhesin gene sequence with publicly available *E. coli* genomes, the adhesin was found to be present among ExPEC strains including APEC and UPEC [14], [15] and is not harboured by non-pathogenic *E. coli* nor by any of the intestinal pathogenic *E. coli* like enteropathogenic *E. coli* (EPEC) or enterohaemorrhagic *E. coli* (EHEC), making it unique to extraintestinal infections. This result therefore validates the temporary name ExPEC adhesin I (EA/I) allotted to the new adhesin.

Fimbrial adhesins share common genetic organization, in that the adhesin regulatory genes precede the major subunit gene, which is followed by the periplasmic chaperone, outer membrane usher, and finally the adhesin genes [10]. This gene cluster organization is seen in type 1 fimbriae (*fim*), S fimbriae (*sfa*), F1C fimbriae (*foc*) and the Dr-antigen recognizing fimbriae (*dra*) [10]. Organization of the *yqi* adhesin gene cluster differs by having the positions of the usher and chaperone inverted, which is also true for the *pap* adhesin gene cluster of the P fimbriae [10], although the reason for this, be it functional or biological, is still unclear.

To date, there are no studies implying the role of ExPEC adhesin I (*yqi*) in the colonization of host tissues during infection. Previously, it has been shown that the *fimH* adhesin of the type 1 fimbriae, and the *papG* adhesin of the P fimbriae are the receptor specific binding proteins that are located at the tip of the pilus structure [28]. It has also been reported that deletion of the *papG* gene had no effect on pilus formation; however, the pili isolated from a *papG* deletion strain were not adhesive [29]. We now have evidence which shows that deletion of the EA/I gene, *yqi*, in APEC strain IMT5155 results in a significant decrease in the colonization of fibroblasts and epithelial cells *in vitro* and of the chicken lung *in vivo*. It is possible that the Yqi adhesin is the receptor-specific protein responsible for initial attachment to host cells; however, this receptor is yet to be identified.

APEC strain IMT5155 has been previously tested for the presence of important adhesins like *fim*, *pap*, *crl*, *sfa*, *tsh*, *afa*, *dra*, *foc* and others, and has been found to harbour only the type 1 fimbria (*fim*), curli (*csg*) and temperature-sensitive haemagglutinin (*tsh*) genes [4]. The absence of P, F1C, S and Dr fimbriae in IMT5155, which are known for their role in UPEC pathogenesis [10], made it apparent that there might in fact be unidentified adhesins that participate crucially during initiation of infection. We observed that in both infection models used in this study, there was always a substantial reduction in the colonization of the chicken lung when infecting chickens with IMT5155Δ*yqi*. Therefore, we believe that ExPEC adhesin I makes a significant contribution to total bacterial adhesion and colonization of the chicken lung by APEC strain IMT5155 during infection. We further observed that there was only a slight reduction in bacterial numbers isolated from internal organs of chickens when infected with IMT5155Δ*yqi* in the systemic infection model. This is not unique, because IMT5155 harbours a number of known virulence genes like iron acquisition genes (*chuA*, *fyuA*, *ireA*, *sitD*), serum resistance genes and protectins (*iss*, *ompA*, *traT*, *neuC*) and invasion related genes (*gimB*, *ibeA*) which play a role in the initiation and prolongation of disease and which may on the whole contribute to systemic infection in chickens [4]. Besides with an infection dose of 10^9^ CFU as used in the systemic infection model, the chicken lung is overwhelmed with bacteria, and therefore, there is very little room left for specific adhesion to take place in contrast to the lung infection model. This could also be a reason for the larger numbers of bacteria in internal organs during systemic infection. However, we still observe a significant bacterial reduction in the lung even with a high infection dose, which suggest the existence of specific receptors for ExPEC adhesin I on the lung epithelium, which account for a significant amount of fimbrial receptors on the lung. Due to the alternative fimbriae still present in the knock-out mutant of ExPEC adhesin I, attachment to other receptors on the surface of the lung would still occur strongly, particularly in the systemic infection model with its high infection dose, thus in turn initiating a systemic infection in this model.

An interesting result was the prevalence of EA/I among a relatively large collection of strains, particularly its high incidence among ExPEC strains like APEC, UPEC, NMEC and septicaemia associated *E. coli* (SePEC) strains, infrequent occurrence among non-pathogenic strains and complete absence among intestinal pathogenic *E. coli* strains. It has been previously reported that distinctive strains of *E. coli* responsible for most cases of urinary tract infection, sepsis and newborn meningitis are derived predominantly from *E. coli* phylogenetic group B2 [30]. Additionally, diverse studies show that virulent clonal groups are derived mainly from phylogenetic group B2 and to a lesser extent from group D [4], [31]. Our observations show that 66.1% of the isolates positive for *yqi* belong to the phylogenetic group B2 in contrast to only 11.5% of isolates negative for *yqi* belonging to this group.

Furthermore, we found that all of the isolates belonging to sequence types 95, 140, 141 and 372 were positive for *yqi*. ST95 and ST140 belong to the sequence type 95 complex. It is known that well defined pathogens are associated with specific STs or ST complexes, for example, ST95 complex contains related pathogenic bacteria of serogroups O1, O2, and O18 that express the K1 polysaccharide, that is, the K1 isolates [24], [26]. Of immense interest, therefore, was the evolutionary analysis of the adhesin gene sequences from strains belonging to different sequence types, which showed that within a particular sequence type complex (STC), the adhesin sequence was homologous which is the case for STC12 and STC73 as well as other sequence types like ST372, ST141 and ST358 with a single exception, strain IMT15008. Since this strain had no unique characteristic that differentiated it from other strains within the ST73 complex, a possible explanation could be that the *yqi* gene in strain IMT15008 is the result of a recombination event.

Interestingly, within the ST95 complex, the adhesin gene sequence showed the presence of single nucleotide polymorphisms (SNPs) which were confirmed as a positive selection on the gene within this complex. Mutations producing functional modifications are called pathogenicity-adaptive or pathoadaptive, and are often SNPs producing amino acid replacements in proteins essential for a pathogen's success and it has been shown that two major adhesins of extraintestinal pathogenic *E.coli* (ExPEC) – type 1 and P fimbrial adhesins – acquire structural SNPs at a rapid rate, and this adaptation constitutes a major factor in the pathoadaptive microevolution and genetic diversification of ExPEC clonal groups [26]. It is possible, that ExPEC adhesin I also undergoes structural mutations or pathoadaptive mutations, particularly, among strains belonging to the ST95 complex, which further confirms the importance of this adhesin within this highly pathogenic complex. Not surprisingly therefore, is the fact that the wild type strain used in this study, IMT5155, also belongs to the ST95 complex and harbours the most number of SNPs in the gene coding for ExPEC adhesin I as can be seen from the branching pattern on the dendrogram.

It therefore makes sense to assume that ExPEC adhesin I could perhaps play a very specific role in ExPEC pathogenesis, especially during the early colonization steps of infection, although this still needs to be proven for UPEC and NMEC strains. However, an interesting addition to this story is the decrease in adhesion to kidney epithelial cells as shown by our *in vitro* adhesion experiments. This would indicate the importance of *yqi* in UPEC, which particularly colonize the bladder epithelium during urinary tract infection (UTI). Furthermore, it provides evidence for the zoonotic potential of APEC, a topic of great interest in the field of ExPEC.

The presence of a single virulence factor hardly ever makes a strain virulent, while a combination of virulence factors usually determines its ability to cause disease [32]. Therefore, much like countless other genes that are classified into the class of virulence factors when exceedingly prevalent among pathogens, the *yqi* gene could also eventually be an addition to this category based on its regular presence in highly pathogenic ExPEC strains in contrast to non pathogenic strains. One must keep in mind, that the meager group of A_faecal_ strains and human non pathogenic strains that were found to be positive for *yqi* in this study are no classical non-pathogenic strains as seen in a previous study [33]. Ewers et al. reported that a number of A_faecal_ *E. coli* strains have characteristics typical of human and animal ExPEC, and that some nonoutbreak strains are capable of causing systemic disease in immunocompetent 5-week old chickens, suggesting the avian intestine reservoir hypothesis [33]. Furthermore it was reported that these strains pose a zoonotic risk because they could be transferred directly from birds to humans or serve as a genetic pool for ExPEC strains. With regard to the non pathogenic human isolates found to be positive for *yqi*, it is interesting to note that though these strains were isolated from the intestinal tract of healthy humans they express the K1 polysaccharide otherwise associated with highly pathogenic strains.

Therefore, taken together, the results of the ExPEC adhesin I (*yqi*) prevalence study in *E. coli* are in fact intriguing, since the search for novel virulence factors still goes on. Nevertheless, they do not imply that EA/I would be solely responsible for virulence of ExPEC strains, rather only contribute to pathogenesis on the whole.

An interesting result in this study was the successful expression of ExPEC adhesin I *in vitro*. We hypothesized that cloning of the putative adhesin gene cluster coding for the putative subunit protein, putative usher and chaperone proteins and putative adhesin, which together are believed to be responsible for expression of fimbrial structures, when successfully cloned in an afimbriate *E. coli* strain would enable the expression of EA/1 fimbriae visible under the electron microscope. Using negative staining and transmission electron microscopy, our hypothesis was indeed confirmed, in that, short fimbrial like structures were detected in the afimbriate strain overexpressed with the adhesin gene cluster, which were not observed in the afimbriate strain, or negative control. The wild type strain IMT5155 used as a positive control revealed long fimbrial structures representing all fimbrial adhesins harboured by the strain including ExPEC adhesin I which explains the difference in length of fimbrial structures observed. The short fimbrial structures observed in the afimbriate strain overexpressed with ExPEC adhesin I will have to be confirmed using specific antibody and immunogold staining in the future; however, this study provides preliminary evidence for the fimbrial structures of this newly identified fimbrial adhesin. ExPEC adhesin I is therefore confirmed to be a novel fimbrial adhesin, worthy of further studies, which will indeed enable a better understanding of the interactions between adhering ExPEC and their host cells.

We have identified an adhesin that plays a significant role in the initial stages of APEC infection. Previous studies with other adhesins, like the *fimH* adhesin, have shown that antibodies elicited against the adhesin can impede colonization, block infection and prevent disease, that is, prophylactic vaccination with adhesins can inhibit bacterial infections [34]. In other studies, it was found that vaccination with Gal-Gal pili or the P fimbrial vaccines prevented pyelonephritis by piliated *E. coli* in a murine model and in monkeys [35], [36]. Therefore, blocking initial stages of infection may be one of the most effective strategies to prevent bacterial infections. Furthermore, it would be interesting to identify the specific receptor to which the EA/I binds. Blocking of specific receptors could also be a means of preventing early infection stages. It is our aim to test ExPEC adhesin I for its vaccine potential in chickens making use of our well established infection models, in order to come one step closer to the final goal of any research in the field of infectious pathogens, namely the prevention of infection and disease.

## Materials and Methods

### Ethics Statement

All animal experiments were approved by the “Landesamt fuer Gesundheit und Soziales” (LAGeSo) (G 0220/06) and chickens were killed according to animal welfare norms (Reg. 0220/06).

### Bacterial Strains, Plasmids and Growth Conditions

All *E. coli* strains and plasmids used are listed in table 3. The wild type APEC isolate IMT5155 (O2:K1:H5) was obtained from internal organs of a laying hen clinically diagnosed with systemic APEC infection during an outbreak in the northern part of Germany in the year 2000 [1], [20]. The strain possesses a number of virulence-associated genes typical of ExPEC strains, among others, curli fibre gene (*csgA*), type 1 fimbriae (*fimC*), temperature-sensitive haemagglutinin (*tsh*), heme receptor gene (*chuA*), outer membrane protein (*ompA*), invasion of brain endothelium (*ibeA*), genetic island associated with newborn meningitis (*gimB*), colicin V plasmid (*cvaC*) and is classified into the B2 phylogenetic group, and multi locus sequence type 140 of the ST95 complex [4]. An additional *E. coli* strain IMT11327 (Ont:H16, ST295, ST23 complex), isolated from the intestine of a clinically healthy chicken, was used as a non-virulent control in both *in vivo* and *in vitro* assays, while *E. coli* - K12 strain (MG1655) was used as a negative control *in vitro*. IMT5155 was used for all genetic manipulation studies. All isolates used in the prevalence studies are part of a strain collection in our laboratory obtained from various sources [4], [33], [37], [38].

**Table 3:** Strains and plasmids used in this study.

Strain	Description	Reference
IMT5155	O2:K1:H5; *csgA*, *fimC*, *tsh*, *chuA*, *fyu*A, *ireA*, *iroN*, *irp2*, *iucD*, *iutA*, *sit*, *neuC*, *ibeA*, *gimB*, *colV*, *ompA*, ST140, STC95, B2	[20]
IMT5155 Nal^R^	IMT5155 derivative, Nalidixin resistant	[20]
S17λpir	*recA thi* pro *hsdR*- M+ RP4::2-Tc::Mu::Km Tn7 lysogenized with λpir phage	[20]
IMT11327	Ont:H16; *fimC*, *crlA*, *ompA*, ST 295, B1	[21]
MG1655	F^-^, Lam^−^, Fim^+^	[44]
EA7F9	IMT5155 Nal^R^ *yqi*::mini-Tn5	this study
IMT5155Δ*yqi*	IMT5155 Nal^R^ Δ*yqi*::Cm^R^	this study
*E. coli* TOP10	F- *mcrA* Δ (*mrr*-*hsd*RMS-*mcr*BC) Φ80*lac*ZΔM15 Δ*lac*X74 *recA1 araD*139 Δ (araleu)7697 *galU galK rpsL* (StrR) *endA1 nupG*	Invitrogen
pCR2.1-TOPO:*yqi*	*E. coli* TOP10 [pCR2.1 TOPO:*yqi*]	this study
pDSK602:*yqi*	*E. coli* TOP10 [pDSK602:*yqi*]	this study
IMT5155Δ*yqi* (pDSK602:*yqi*)	IMT5155 Nal^R^ Δ*yqi*::Cm^R^ [pDSK602:*yqi*]	this study
IMT5155Δ*yqi* (pDSK602)	IMT5155 Nal^R^ Δ*yqi*::Cm^R^ [pDSK602]	this study
IMT5155 (pDSK602:*yqi*)	IMT5155 Nal^R^ [pDSK602:*yqi*]	this study
pCR2.1-TOPO:*yqi*_operon	*E. coli* TOP10 [pCR2.1 TOPO:*yqi*-5kb]	this study
Top10 (pKESK:*yqi*_4975_ XB)	*E.coli* TOP10 [pKESK-22:*yqi*_4975 bp_*XbaI*-*BamHI*]	this study
*E. coli* AAEC189	Δfim, Δlac, recA-, endA-, hsdR-, hsdM+	[45]
*E. coli* AAEC189 (pKESK: *yqi*_4975_XB)	*E.coli* AAEC189 [pKESK-22:yqi_4975 bp:XbaI-BamHI]	this study
**Plasmid**		
pUTmini-Tn5km2	Kan^R^, Amp^R^	[20]
pKD46	Amp^R^, expresses λ red recombinase	[39]
pKD3	*cat* gene	[39]
pCR2.1 TOPO	Kan^R^, Amp^R^, LacZα, T7 promoter	Invitrogen
pKESK-22	Neo^R^, Kan^R^, *tac* promoter	K. Schnetz (Uni Köln)
pDSK602	Spec^R^, Sm^R^, triple *lac* UV5, broad host range	[40]

Bacterial strains were routinely cultured at 37°C in Luria-Bertani (LB) broth and on LB agar plates with appropriate antibiotics when required in the following concentrations: Kanamycin (Kan), 50 µg/ml; Nalidixin (Nal), 30 µg/ml; Ampicillin (Amp), 50 µg/ml; Chloramphenicol (Cm), 30 µg/ml; Spectinomycin (Spec) 50 µg/ml. All strains were stored at −70°C in LB broth with 10% (v/v) glycerol until further use.

### PCR Analyses

All oligonucleotide primers are listed in table 4. Unless otherwise specified, *E. coli* isolates, from which DNA was to be used as the template for PCR amplification, were grown overnight in Luria-Bertani broth at 37°C and DNA was released from whole organisms by boiling for 10 minutes. After centrifugation, 2 µl of the supernatant was taken as template DNA and added to a 25 µl reaction mixture containing 0.1 µl of each primer pair in a 10 pmol concentration, 0.3 µl 10 mM of the four deoxynucleoside triphosphates (Sigma-Aldrich Chemie GmbH, Munich Germany), 2.5 µl of 10x PCR buffer, 1 µl of 50 mM magnesium chloride and 1 unit of *Taq*-Polymerase (Rapidozym GmbH, Berlin Germany). The samples were subjected to 25 cycles of amplification in a thermal cycler (GeneAmp PCR system, Applied Biosystems, Darmstadt, Germany). The amplification products were analyzed by gel electrophoresis on a 1.5% agarose gel (Biodeal, Markkleeberg, Germany), stained with ethidium bromide and photographed on exposure to UV.

**Table 4:** Oligonucleotide primers used in this study.

Primer number	Target region	Primer Sequence (5′–3′)	Tm (°C)	Reference
IMT-P2510	*cat* (pKD3)	AATCCCTCTGCCAAAGCTCTCCTGCTAAGAAGGGGAAAATGTGTAGGCTGGAGCTGCTTCGA	75	this study
IMT-P2511	*Cat* (pKD3)	TAAATATGAAAATGCCGGGGTGTTCCCGGCATTTTGCTGTCATATGAATATCCTCCTTAG	71	this study
IMT-P2512	*Yqi*	ATGCAATGGCAGTACCCTTC	60	this study
IMT-P2513	*Yqi*	CTGGTGGCAACATCAAATTG	60	this study
IMT-P2558	221 bp upstream of *yqi*	ATTACCGTCGGTTATATCGGC	52	this study
IMT-P2559	110 bp downstream of *yqi*	ATAAACACAATATGGCGCTCG	50	this study
IMT-P2910	*yqi* with *EcoRI*	CGGATACGAATTCATGATTACGCTTTTTCGTT	59	this study
IMT-P2911	*yqi* with *HindIII*	TTCTCAAAAGCTTTGTCGTTCAGTTATAGTTTA	57	this study
IMT-P3045	*yqi* operon with *BamHI*	CGGATACGGATCCATGTTAAAAAAAACATTGTTATCTATGTTCGCAAC	56	this study
IMT-P3046	*yqi* operon with *HindIII*	TTCTCAAAAGCTTTCAGTTATAGTTTATTTTTACGATGAGATC	53	this study
IMT-P1560	Cloning vector pCR2.1 TOPO (−20)	GTAAAACGACGGCCAG	50	Invitrogen
IMT-P1561	Cloning vector pCR2.1 TOPO	CAGGAAACAGCTATGAC	50	Invitrogen
IMT-P718	*Cat*	TTATACGCAAGGCGACAAGG	57.3	[20]
IMT-P719	*Cat*	GATCTTCCGTCACAGGTAGG	59.4	[20]
IMT-P3138	Expression vector pKESK-22	AATGTGTGGAATTGTGAGCGG	60.6	this study
IMT-P3139	Expression vector pKESK-22	GCCGACATGATCCAACTGA	60.1	this study
IMT-P3706	*Yqi*	AGTTAGGCTTTGTGGCGGAC	56.3	this study
IMT-P3707	*Yqi*	GTTCACCGTCTATCTCCT	53.6	this study
IMT-P3259	*yqi* operon with *XbaI*	CGGTACTCTGAATGTTAAAAAAAACATTGTTATCTATGTTCGCAAC	67.3	this study
IMT-P3260	*yqi* operon with *BamHI*	TTCTCGATCCTCAGTTTAGTTTATTTTTACGGGATC	66.6	this study

### Establishment of a Lung Colonization Model of Infection

A modified lung colonization model of infection was established based on the existing systemic infection model, for the purpose of screening STM mutant pools for adhesion and colonization factors as described previously according to animal welfare norms [21]. The lung infection model differed from the systemic infection model in that, the infection dose was lowered to 10^6^ CFU such that no systemic infection was induced.

Briefly, five-week old white leghorn specific pathogen free (SPF) chickens (Lohmann Tierzucht GmbH, Cuxhaven, Germany) were used for infection purposes. Groups of 10 chickens were infected intra-tracheally with a 0.5 ml suspension containing 10^6^, 10^5^, 10^4^ and 10^3^ CFU of the virulent strain IMT5155 respectively. IMT11327 was used as a negative control during infection. Chickens were euthanized 24 h post infection, and a clinical score was determined which monitored the infection ranging from score 0 (no symptoms) to score 4 (severe symptoms). An organ lesion score was likewise determined with a minimum and maximum score of 1 and 5 in the lungs respectively depicting the severity of lesions in the lungs, and a score of 0 (no hyperplasia) or 1 (hyperplasia) in the spleen. Bacteria were isolated from the lungs and spleen as follows: Organ samples were weighed, suspended in phosphate-buffered saline (1 ml/g) and homogenized with an Ultra-Turrax apparatus. Serial dilutions were plated out onto LB agar plates which were then incubated at 37°C for 24 h. Colonies were then counted to determine the CFU per gram in each organ. An infection dose of 10^6^ was made the infection dose of choice in subsequent infections, since the number of re-isolated bacteria with this infection dose was optimal for STM analyses (data not shown).

### Transposon pUTmini-Tn5 km2 Mutagenesis and Identification of a Novel Adhesin

Insertion mutagenesis of APEC strain IMT5155 was performed randomly by using transposon pUTmini-Tn5*km2* as previously described [20]. A transposon mutant library of strain IMT5155 was generated in this study and screened as described previously by Li et al [20], in the chicken lung colonization model of infection in search for colonization and adhesion factors crucial to APEC infection. A mutant EA7F9, was selected in this screen and the disrupted gene *yqi*, encoding a putative adhesin, was identified using arbitrary PCR also described previously [20]. This adhesin, temporarily renamed ExPEC adhesin I (EA/I) was further characterized for its role in APEC pathogenesis.

### Functional Characterization of ExPEC Adhesin I (EA/I)

#### *In vitro* and *in vivo* competition assays

*In vitro* and *in vivo* competition assays were performed by mixing cultures of mutant and wild type strains with an OD_600_ = 1 in a ratio of 1∶1. For *in vitro* assays, the bacterial mixture was incubated in LB broth for 4 h at 37°C and then plated onto LB plates with and without Kanamycin and Nalidixin. For *in vivo* assays, four chickens were infected with 10^6^ CFU/ml of this bacterial mixture. At 24 h post infection chickens were euthanized, lungs homogenized and serial dilutions plated onto LB plates with and without Kanamycin and Nalidixin for selection of the mutant and wild type strain respectively. A competitive index (CI) was calculated by dividing the output ratio (CFU mutant: CFU wild type) by the input ratio (CFU mutant: CFU wild type) at 0 h and 4 h or 24 h for *in vitro* and *in vivo* assays respectively.

#### Sequencing of a 4,975 bp EA/1 gene cluster region in IMT5155

In order to sequence the EA/I gene cluster (*yqi*) in IMT5155, the 4,975 bp region was amplified using primers IMT-P3045 and IMT-P3046 and the PCR product was cloned into pCR2.1 TOPO vector according to the standard TOPO cloning manual (Invitrogen GmbH, Karlsruhe, Germany). The cloned product was transformed into *E. coli* TOP10 by electroporation and plated out on LB with Kanamycin to select for positive clones. Colonies were tested for the presence of the *yqi* gene cluster (4,975 bp) using standard primers IMT-P1560 and IMT-P1561. The plasmid containing the insert was isolated from the host strain and sequenced commercially by LGC's AGOWA Genomics, Berlin, Germany.

#### Generation of an isogenic *yqi* mutant

To generate a knock-out mutant of the fimbrial adhesin in IMT5155, the *yqi* gene was replaced with a Chloramphenicol resistance cassette using the lambda red recombinase system [39]. The Chloramphenicol acetyl transferase (CAT) gene was amplified from plasmid pKD3 using PCR with primers IMT-P2510 and IMT-P2511 (Table 4), part of which have sequence homology to the *yqi* flanking region. The PCR product was purified on a 1% agarose gel and 2 µl of the sample was transformed by electroporation into IMT5155 containing the lambda red recombinase expression plasmid pKD46. After electroporation, samples were incubated at 28°C for 1 h in SOC broth and plated on LB agar with Chloramphenicol to select for positive clones (CAT). After overnight incubation at 37°C, transformants were selected and tested for loss of the *yqi* gene using PCR with primer pairs IMT-P718/IMT-P2558 and IMT-P719/IMT-P2559 (Table 4).

#### Complementation of *yqi*

For complementation and over-expression studies the IMT5155 *yqi* gene (1050 bp) PCR product was cloned into pCR2.1 TOPO vector according to the standard TOPO cloning manual (Invitrogen GmbH, Karlsruhe, Germany) using primers IMT-P2910 and IMT-P2911 (Table 4) with restriction enzyme recognition sites *EcoRI* and *HindIII*, and transformed into *E. coli* TOP10 via electroporation. Positive colonies were selected on LB agar with Kanamycin and tested for the presence of *yqi* using standard primers IMT-P1560 and IMT-P1561 (Table 4). The TOPO vector with the IMT5155 *yqi* insert was commercially sequenced by LGC's AGOWA Genomics, Berlin, Germany to determine that the sequence was in frame. The pCR2.1 TOPO:*yqi* vector and expression vector pDSK602 [40] were then digested with restriction enzymes *EcoRI* and *HindIII* for 1 h at 37°C, and ligated using T4 DNA ligase overnight at 4°C. Three microliters of the ligation mix were then electroporated into *E. coli* TOP10 and plated out on LB agar containing spectinomycin. Colonies were tested for the presence of *yqi* using PCR with primers IMT-P2910 and IMT-P2911 (Table 4). The modified plasmid pDSK602 with the *yqi* insert was isolated from *E. coli* TOP10 and transformed into IMT5155Δ*yqi* to complement the gene deleted. In adhesion and colonization assays, the complemented strain was induced with IPTG in a final concentration of 100 mM for expression of the protein.

#### Adhesion assays *in vitro* with Chicken Fibroblast (CEC) and polarized Madin Darby Canine Kidney (MDCK-1) epithelial cell lines

Chicken fibroblast (CEC-32) cells [41] were used between passages 6 and 9 and were seeded into 12-well microtitre plates at a density of ∼2×10^5^ cells per well and incubated at 37°C in an atmosphere containing 5% CO_2_ without antibiotics prior to adhesion assays. Minimal essential cell culture medium (Pan^TM^ Biotech GmbH, Aidenbach, Germany) with 5% Foetal calf serum (FCS) (Pan^TM^ Biotech GmbH, Aidenbach, Germany) was used to grow cells. Monolayers were used after 6 days incubation.

Madin Darby Canine Kidney (MDCK-1) [42] cells were used between passages 1 and 5. Cells were grown in Dulbecco's modified eagle's medium (DMEM) (Pan^TM^ Biotech GmbH, Aidenbach, Germany) with 10% FCS and were incubated at 37°C in 5% CO_2_. Transwell filter units (Costar) (Sigma-Aldrich Chemie GmbH, Munich Germany) contained a 1.12 cm^2^ porous filter membrane (0.4-µm pores) that had been treated for tissue culture. Filter units were incubated in 12-well microtitre plates (Costar) and were placed in DMEM containing 10% FCS for 1 h, at 37°C before seeding. 40 µl of a trypsinized MDCK cell suspension were added to each Transwell unit. Monolayers were used after 4 days incubation at which time there were around ∼3×10^5^ MDCK cells per filter.

For adhesion assays, bacteria were added to the appropriate wells in triplicate in medium without FCS at an MOI of 100, that is, ∼2–3×10^7^ bacteria per well. Microtitre plates were centrifuged at 250 x *g* for 10 minutes, and then incubated for 1.5 h and 3 h for CEC cells, and 3 h for MDCK cells, after which the supernatant was discarded; cells were washed thrice with Phosphate buffered saline (PBS) and then plated out on LB agar to determine the number of adherent bacteria in each well.

#### Animal infection studies

Animal experiments were also carried out to determine the colonization ability of strain IMT5155Δ*yqi* in comparison to IMT5155. IMT11327 was used as the negative control. Briefly, in an assay involving the lung colonization chicken model, groups of 6 five-week old chickens each were infected intratracheally with a bacterial suspension containing 10^6^ bacteria per ml. At 24 h post infection, chickens were euthanized and dissected. A clinical and organ score was recorded and the lungs and spleen were homogenized.

In the chicken systemic infection model, groups of 6 five-week old chickens were infected intratracheally with a bacterial suspension containing 10^9^ bacteria per ml. At 24 h post infection chickens were euthanized, dissected and a clinical and organ score was recorded. The lungs, heart, liver, kidneys, spleen and brain were homogenized.

All homogenates were appropriately diluted and plated out on LB agar and LB agar with antibiotics where required, to determine the number of bacteria colonizing the chicken lung and number of bacteria in internal organs during systemic infection.

#### Over-expression of the 4,975 bp EA/I adhesin gene cluster in fimbrial negative *E. coli* strain AAEC189

The 4,975 bp *EA/1* adhesin gene cluster was amplified and the PCR product cloned into pCR2.1 vector according to the standard TOPO cloning manual (Invitrogen GmbH, Karlsruhe, Germany) using primers IMT-P3259 and IMT-P3260 with restriction enzyme cutting sites *XbaI* and *BamHI*, and transformed into electro-competent *E. coli* TOP10 cells via electroporation. Positive clones were selected on LB agar with Kanamycin and tested for the presence of the 4,975 bp gene cluster using standard primers IMT-P1560 and IMT-P1561.

The plasmid pCR2.1-TOPO:*yqi*_4975_XB and expression vector pKESK-22 were digested with restriction enzymes *XbaI* and *BamHI*, and ligated using T4 DNA ligase overnight at 4°C and finally electroporated into electro-competent *E.coli* TOP10 cells and plated out on LB agar containing spectinomycin. Colonies were tested for the presence of the 4,975 bp adhesin gene cluster using PCR with primers IMT-P3138 and IMT-P3139.

Plasmid pKESK:*yqi*_4975_XB was isolated from *E. coli* TOP10 and transformed into electro-competent *E. coli* AAEC189 (afimbriate *E. coli* strain) cells, to over-express the adhesin gene cluster.

#### Electron microscopy

*E. coli* strains AAEC189, AAEC189 (pKESK:*yqi*_4975_XB) and IMT5155 were grown in 5 ml Brain Heart infusion broth after inoculating with 150 µl of the respective overnight cultures. Strains AAEC189 and IMT5155 were used as negative and positive controls respectively. Strain AAEC189 (pKESK:*yqi*_4975_XB) was additionally given Kanamycin in its growth medium, and induced with 0.1 M IPTG after 30 minutes of growth. All three strains were grown to an OD_600_ of 2.5, allowing an induction time of 2.5 h for strain AAEC189 (pKESK:*yqi*_4975_XB). One millilitre of bacterial culture was centrifuged at 8000 x *g* and the bacterial pellet was washed thrice with 1x PBS and then resuspended in 500 µl 1x PBS. Twenty microlitres of bacterial suspension were applied to Formvar-coated 200- mesh copper grids and stained with 1% Uranyl acetate for 2 mins. Negatively stained preparations were examined on a Zeiss EM900 microscope.

#### Prevalence of the EA/1 gene *yqi* among ExPEC, intestinal pathogenic *E. coli* (IPEC) and commensals

To determine the prevalence of *yqi* among *E. coli* strains, 406 avian pathogenic *E. coli* (APEC) strains, 138 uropathogenic *E. coli* (UPEC), 25 Newborn meningitic *E. coli* (NMEC), 19 Septicaemia associated *E. coli* (SePEC), 153 intestinal pathogenic *E. coli* (IPEC) and 159 non pathogenic strains, including faecal strains from clinically healthy chickens (A_faecal_) and non pathogenic strains isolated from the intestinal tract of healthy humans, were tested for the presence of the *yqi* gene using standard PCR reactions with primers IMT-P2512 and IMT-P2513 by way of amplification of a 400 bp region of the adhesin gene as described under PCR analyses. IMT5155 was used as a positive control, and IMT11327 as a negative control for all PCR reactions. Results were observed as a single clear band on an agarose gel and recorded as positive and negative for all strains respectively.

#### Sequencing of the EA/1 gene *yqi* and evolutionary analysis

A total of 77 strains representing multi locus sequence types (MLST) ST12, ST73, ST95, ST104, ST135, ST140, ST141, ST358, ST363, ST368, ST372, ST390, ST416, ST417 and ST421 were selected for sequencing of the *yqi* gene (1050 bp), from a previous study at the Institute of Microbiology and Epizootics involving MLST analysis, using primer pairs IMT-P3706/IMT-P3707 and IMT-P2512/IMT-P2559. The PCR products were sequenced commercially by LGC's AGOWA Genomics, Berlin, such that a double stranded sequence was obtained for each strain. Sequences were analysed using Kodon software available from Applied Maths. A phylogenetic tree showing distances between strains of different STs was calculated by a maximum parsimony algorithm using Kodon Software from Applied Maths. Bootstrap values were computed with 100 replicates. The adhesin gene sequence of the strains APEC_O1 (APEC) (Acc. No: CP000468), CFT073 (UPEC) (Acc. No: AE014075), UTI89 (UPEC) (Acc. No: CP000243) was obtained from the available nucleotide database, and compared with sequenced strains in this study. Rates of non-synonymous (Dn) and synonymous (Ds) mutation were calculated using DnaSP 4.50.3 software [43] for the sequenced adhesin gene among strains belonging to the ST95 complex in order to determine the Dn/Ds ratio for each locus as described previously [26].

### Statistical Analysis

All statistical analysis for *in vivo* animal experiments and *in vitro* cell culture experiments were carried out using the software SPSS (Statistical package for the social sciences), version 15.0 by carrying out the non-parametric Mann-Whitney U-Test and the students *t*-test at the 95% significance level (*p*<0.05).
